# Wndchrm – an open source utility for biological image analysis

**DOI:** 10.1186/1751-0473-3-13

**Published:** 2008-07-08

**Authors:** Lior Shamir, Nikita Orlov, D Mark Eckley, Tomasz Macura, Josiah Johnston, Ilya G Goldberg

**Affiliations:** 1 Image Informatics and Computational Biology Unit, Laboratory of Genetics, NIA/NIH, 333 Cassell Dr., Baltimore, MD, 21224, USA; 2 Computer Laboratory, University of Cambridge, 15 Thomson Avenue, Cambridge, UK; 3 Energy and Resources Group, University of California Berkeley, 1519 Addison St., Berkeley, CA, 94720-3050, USA

## Abstract

**Background:**

Biological imaging is an emerging field, covering a wide range of applications in biological and clinical research. However, while machinery for automated experimenting and data acquisition has been developing rapidly in the past years, automated image analysis often introduces a bottleneck in high content screening.

**Methods:**

*Wndchrm *is an open source utility for biological image analysis. The software works by first extracting image content descriptors from the raw image, image transforms, and compound image transforms. Then, the most informative features are selected, and the feature vector of each image is used for classification and similarity measurement.

**Results:**

*Wndchrm *has been tested using several publicly available biological datasets, and provided results which are favorably comparable to the performance of task-specific algorithms developed for these datasets. The simple user interface allows researchers who are not knowledgeable in computer vision methods and have no background in computer programming to apply image analysis to their data.

**Conclusion:**

We suggest that *wndchrm *can be effectively used for a wide range of biological image analysis tasks. Using *wndchrm *can allow scientists to perform automated biological image analysis while avoiding the costly challenge of implementing computer vision and pattern recognition algorithms.

## Background

In the past few years, pipelines providing high-throughput biological imaging have been becoming increasingly important, and are supported by automated microscopy and high-performance computing and storage devices. Applications include profiling drug responses, screening for small molecules, classification of subcellular localization, and more. While image acquisition systems, network bandwidths, and storage devices are capable of supporting vast pipelines of biological images, human analysis can be impractically slow. Therefore, machine vision algorithms for analysis of biological images are necessary to complete a fully automated process for interpreting biological experiments.

Some highly useful applications have been proposed and made available to the scientific community. OME [1,2], as well as its derivative product OMERO [3], provide a widely used enterprise solution to storing, handling and processing biological and medical images. Another useful application is CellProfiler [4], which is a tool designed specifically for analyzing microscopy images of cells. Other tools focus on single tasks in the process of biological image analysis. An example is the utility FindSpots, which detects objects (e.g., cells) in an image based upon their intensity and size.

In this paper we present *wndchrm *– an open source utility for biological image analysis that can be used for a wide range of biological datasets, which include organelles, cells, tissues, and full organisms. Using *wndchrm *does not require knowledge in computer vision or computer programming, allowing biologists who do not have strong programming skills to enhance their experiments with automated image analysis capabilities. The code can be downloaded freely via the internet, and does not require any licensed commercial software. It can also be easily integrated into existing or new software products.

## Description of the Method

The image analysis method works by applying a first step of computing a large number of different image features. For image feature extraction we use the following algorithms, described more thoroughly in [5]:

1. **Radon transform features **[6], computed for angles 0, 45, 90, 135 degrees, and each of the resulting series is then convolved into a 3-bin histogram, providing a total of 12 image features.

2. **Chebyshev Statistics **[7] – A 32-bin histogram of a 1 × 400 vector produced by Chebyshev transform of the image with order of N = 20.

3. **Gabor Filters **[8], where the kernel is in the form of a convolution with a Gaussian harmonic function [9], and 7 different frequencies are used (1,2...,7), providing 7 image descriptor values.

4. **Multi-scale Histograms **computed using various number of bins (3, 5, 7, and 9), as proposed by [10], providing 3+5+7+9 = 24 image descriptors.

5. **First 4 Moments**, of mean, standard deviation, skewness, and kurtosis computed on image "stripes" in four different directions (0, 45, 90, 135 degrees). Each set of stripes is then sampled into a 3-bin histogram, providing 4 × 4 × 3 = 48 image descriptors.

6. **Tamura texture features **[11] of *contrast, directionality *and *coarseness*, such that the coarseness descriptors are its sum and its 3-bin histogram, providing 1+1+1+3 = 6 image descriptors.

7. **Edge Statistics features **computed on the Prewitt gradient [12], and include the mean, median, variance, and 8-bin histogram of both the magnitude and the direction components. Other edge features are the total number of edge pixels (normalized to the size of the image), the direction homogeneity [13], and the difference amongst direction histogram bins at a certain angle *α *and *α *+ *π*, sampled into a four-bin histogram.

8. **Object Statistics **computed on all 8-connected objects found in the Otsu binary mask of the image [14]. Computed statistics include the Euler Number [15], and the minimum, maximum, mean, median, variance, and a 10-bin histogram of both the objects areas and distances from the objects to the image centroid.

9. **Zernike features **[16] are the absolute values of the coefficients of the Zernike polynomial approximation of the image, as described in [13], providing 72 image descriptors.

10. **Haralick features **[17] computed on the image's co-occurrence matrix as described in [13], and contribute 28 image descriptor values.

11. **Chebyshev-Fourier features **[18] – 32-bin histogram of the polynomial coefficients of a Chebyshev-Fourier transform with highest polynomial order of N = 23.

Since image features extracted from transforms of the raw pixels can also be informative [5], image content descriptors are extracted not only from the raw pixels, but also from several transforms of the image and transforms of transforms. The image transforms are FFT, Wavelet (Symlet 5, level 1) two-dimensional decomposition of the image, Chebyshev transform, and Edge transform, which is simply the magnitude component of the image's Prewitt gradient, binarized by Otsu global threshold [14].

In the described image classification method, different image features are extracted from different image transforms or compound transforms. The software allows the user to choose between extracting a smaller set of image features, which includes 1025 content descriptors, and extracting a larger set of 2659 features. The larger set of image features can be more informative, but also requires the sacrifice of more computational resources, which leads to a slower response time. The image features extracted from each transform in the smaller and larger feature sets are described by Table 1.

**Table 1:** Image features

Image Features											
Transform	Radon	Chebyshev statistics	Gabor	Multi-scale histograms	First 4 moments	Tamura texture	Edge statistics	Object statistics	Zernike	Haralick	Chebyshev-Fourier

Raw Pixels	Both	Both	Both	Both	Both	Both	Both	Both	Both	Both	Both
FFT	Both	Both	None	Both	Both	Both	None	None	Both	Both	Both
Chebyshev	Both	Large	None	Both	Both	Both	None	None	Large	Both	Large
Wavelet	Large	Large	None	Both	Both	Both	None	None	Large	Both	Large
FFT-Chebyshev	Both	Large	None	Both	Both	Both	None	None	None	Both	None
FFT-Wavelet	Large	Large	None	Both	Both	Both	None	None	None	Both	None
Edge	Large	Large	None	Large	Large	Large	None	None	Large	Large	Large
Edge-Fourier	Large	Large	None	Large	Large	Large	None	None	Large	Large	Large
Edge-Wavelet	Large	Large	None	Large	Large	Large	None	None	Large	Large	Large
Chebyshev-FFT	Large	None	None	Large	Large	Large	None	None	None	Large	None
Wavelet-FFT	Large	None	None	Large	Large	Large	None	None	None	Large	None

The image features extracted from the different image transforms in the smaller and larger feature sets. For each type of image feature extracted from each transform the table specifies whether these features are extracted in the smaller feature set, larger feature set, both sets, or none of the sets.

While the set of image features provides a numeric description of the image content, not all image features are assumed to be equally informative for each dataset, and some of these features are expected to represent noise. To select the most discriminative features and reject the noisy features, each image content descriptor is assigned with a simple Fisher score [19] and rank ordered, so that only the features with the highest Fisher scores are included in the following analysis. This filtering of the image features can be performed only after all image features are computed. In the described software, Fisher scores are computed before test samples are classified.

The feature vectors of given test samples are classified by a variation of nearest neighbor classification. For feature vector *x *computed from a test image, the distance *d*_*x, c *_of the image from a certain class *c *is measured by Equation 1

(1) $$d_{x,c}=\frac{\sum_{t\in T_{c}}[\sum_{f=1}^{\left|x\right|}W_{f}{\left(X_{f}-t_{f}\right)}^{2}{]}^{p}}{\left|T_{c}\right|}$$

where *T*_*c *_is the training set of class *c*, t is a feature vector from *T*_*c*_, |*x*| is the length of the feature vector *x*, *x*_*f *_is the value of image feature *f *in the vector *x*, *W*_*f *_is the Fisher score of feature *f*, |*T*_*c*_| is the number of training samples of class *c*, and *p *is the exponent, which is set to -5. The -5 value has been determined empirically, and is thoroughly discussed in [5]. The distance between a feature vector and a certain class is the mean of its weighted distances (to the power of *p*) to all feature vectors of that class. After the distances from sample *x *to all classes are computed, the class that has the shortest distance from the given sample is the classification result. A detailed description and performance evaluation of this method can be found in [5].

## Image Similarity

Once a classifier is trained, test images can be mapped into the image feature space. The classification of each feature vector provides a vector of the size *N *(N is the total number of classes), such that each entry *c *in the vector represents the similarity of the feature vector to the class *c*, computed by Equation 2,

(2) $$M_{f,c}=\frac{1}{d_{f,c}\cdot \sum_{i=1}^{N}\frac{1}{d_{f,i}}}$$

where *M*_*f, c *_is the computed similarity of the feature vector *f *to the class *c*, and *d*_*f, c *_is the distance computed by Equation 1. This assigns each image in the test set with *N *values within the interval [0, 1], representing its similarity to each class.

Averaging the similarity vectors of all images of a certain class provides the similarities between that class and any of the other classes in the dataset. Repeating this action for all classes results in a full set of similarities between any pair of classes, which can be presented in the form of a similarity matrix. The similarity matrix contains two similarity values for each pair of classes. I.e., the cell *n*, *m *is the similarity value between class *n *to class *m*, and the cell *m*, *n *is the similarity of class *m *to class *n*. Although these two values are expected to be close, they are not expected to be identical due to the different images used when comparing *n *to *m *and *m *to *n*. Averaging the two values provides a single nominal distance between each pair of classes, which can be used for visualizing the class similarity in an intuitive fashion using phylogenies (evolutionary trees) inferred automatically by using the Phylip package [20], as will be described in this paper.

## Implementation

The software is written in C++, and uses the freely available fftw [21] and libtiff libraries. The code is organized such that the feature extraction and pattern recognition algorithms are compiled into a library called "libimfit", so that it can be easily integrated into other software products. The *wndchrm *utility does not have an integrated graphical user interface, and all user interactions are performed using simple command-line instructions. The command line is implemented by the wrapper file "wndchrm.cpp", which uses the functions implemented in *libimfit*.

To allow multi-platform support, the code uses only standard C function calls and avoids using machine-specific APIs. The code is compiled using the standard *gcc *compiler, and can therefore be easily compiled under commonly used operating systems such as OS X, Linux, Unix, etc. Since the source code is fully open, users may also compile it using other compilers such as Borland C++. This can make it easier for advanced users who wish to modify the code for their specific needs or integrate the source files with an existing application. For compiling the code under MS-Windows without using *gcc*, it is advised to define the constant "WIN32". Windows users can also download and use a binary executable file of *wndchrm*.

Many computer vision experts develop their software using MATLAB, and a substantial amount of code and utilities have been written using that tool. Those who wish to integrate *wndchrm *into their existing MATLAB code can use MATLAB's MEX files, which are designed specifically for calling C (and FORTRAN) functions from a MATLAB application.

An important advantage of implementing *wndchrm *in C++ is speed, especially when compared to MATLAB. For example, a MATLAB implementation of the smaller feature set (with 1025 image features) is ~4 times slower than the C++ implementation of the same set. This difference can be highly significant when the dataset is large. Another matter that should be taken into considerations when using MATLAB is licensing. This issue can become crucial if the computation is performed by clusters, where each node of each cluster requires its own license.

## Computational Complexity and Robustness

An obvious downside of *wndchrm *is its computational complexity. Since *wndchrm *works by applying a chain of feature extraction algorithms, the theoretical computational complexity is equal to that of the most expensive algorithm in the chain. This algorithm is the Zernike feature extraction, which has a factorial complexity in terms of its radial polynomial coefficients. However, since many of the other feature extraction algorithms in the chain are also computationally intensive, a very large constant is hidden inside the theoretical complexity of *wndchrm*. Theoretical space complexity is linear to the image size, with a large constant of 100 MB used for computing the Chebyshev-Fourier features.

**Figure 1:**
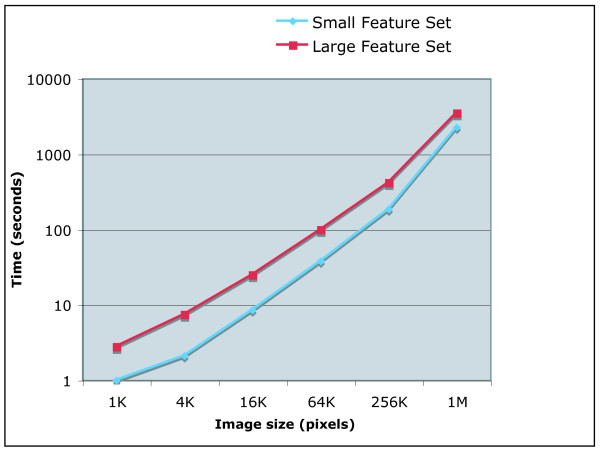
**Time required for processing a single image**. The time required for computing image features for a single image is linear to the number of pixels for images no larger than 512 × 512 pixels.

Practically, the set of image features extracted from the raw pixels and the several transforms require significant CPU resources. For example, the large set of image features can be extracted from one 256 × 256 image in ~100 seconds using a system with a 2.6 GHZ AMD Opteron and 2 GB of RAM. Computing the smaller feature set requires ~38 seconds. The time required for processing a single image increases linearly with the number of pixels, as shown by Figure 1. However, since memory access can become slower when large amounts of memory are used, performance suffers considerably above a certain image size. This can be evident by the sharp decrease in response time when the image size gets larger than 512 × 512 pixels. In these cases, users may consider breaking very large images into tiles, as will be described later in the paper. Since the images are processed sequentially, the overall response time is linear to the number of images in the dataset.

In the sense of memory usage, *wndchrm *stores the original image in the main memory along with the 10 image transforms (when the larger feature set is used). Since each pixel requires 11 bytes (eight bytes for the intensity and one additional byte for each of the three color channels), the total memory allocated for the image is 11 × 11 × width × height bytes. Computing the Chebyshev-Fourier features requires additional 100 MB. Once image features are extracted from an image, all memory blocks are freed, so that the memory used by *wndchrm *does not grow when more images are processed.

The response time of computing the image features can be reduced significantly by running several instances of *wndchrm*, each on a different processor, so that image features of different images are computed simultaneously by different processors. To implement this approach, the feature values of each image are stored in a file, which is opened exclusively by the instance of *wndchrm *that processes that image. If the file already exists, *wndchrm *skips that image and moves on to the next image in the dataset. Another advantage of storing the values of the content descriptors of each image in a separate file is that if the image feature computation is stopped for any reason before completion, restarting the process does not require re-computing image features of images that have already been processed. This aspect of feature extraction can be exploited if more data become available. If new images are added to a dataset that has already been computed, the user can compute image features for the newly added data without re-computing features for the existing images. Activating this feature using the command-line user interface is explained in the next section.

*Wndchrm *has been tested for robustness using a dataset of ~45,000 256 × 384 images, computed using four 2.6 GHZ Opteron quad-core servers (total of 16 processors) with 2 GB of RAM per core. The 16 *wndchrm *instances worked for 8 days with no failure, and the memory allocated for each instance in the last day was equal to the that of day one, indicating on the absence of memory leaks.

## Using the Command Line Utility

While the source code can be easily integrated into existing or new software products, the software tool described in this paper can be used in the form of a command line utility. This allows researchers who do not have programming skills to apply image analysis to their data.

Supervised machine learning classification consists of two primary steps: *Training *a classifier with a set of samples that are considered as "ground truth" data, and *testing *the effectiveness of the classifier using a second set of samples such that none of the samples of the test set are also used for training.

In order to train an image classifier, the first required task is computing image content descriptors for all images in the dataset. These numeric values describe the image content in a fashion that can later be processed by pattern recognition methods. This step is performed by using a simple command line described below:

% wndchrm train [options] *images feature_file*

where *feature_file *is the resulting output file of the image feature values, *images *is a path to the top folder where the images of the dataset are stored, and [*options*] are optional switches that can be specified by the user. The top folder should consist of several sub-folders such that each sub-folder contains images of a different class. The image formats that are currently supported are TIFF and PPM. Since the current version of *wndchrm *does not support three-dimensional image features, multi-sliced tiffs are not supported at this point.

The single output file *feature_file *contains features for all classes in the dataset, so that there are no separate files for the different classes. Therefore, if a new class is added to the dataset, a new file needs to be created using the same command line. To avoid re-computing classes that have already been computed, the user is advised to use the "-m" switch that will be described later in this section.

Once all image content descriptors are computed, the dataset can be tested for classification accuracy. This can be done by using the following command line:

% wndchrm test [options] *feature_file *[report_file]

where *feature_file *is the output file of the train task, and *report_file *is an optional html file providing detailed information regarding the performance of the classifier. This instruction automatically splits the images of each class into training and test images, and the effectiveness of the classifier is determined by the percentage of test images that are classified correctly using the training images. The test images are classified by computing the Fisher scores and assigning the image features with weights. The output of this command is a confusion matrix, a similarity matrix, and the accuracy of the classifier (the percentage of test images that were classified correctly).

After a classifier is trained and tested, an image can be classified using the command line:

% wndchrm classify *feature_file image*

where *image *can be a full path to the image being classified, or a folder that contains multiple *images*. If image points to a specific image file, the output of this instruction is the predicted class in which the image belongs, as well as a vector of similarity values to each of the classes in the dataset. If *image *is a path to a folder, *wndchrm *classifies and prints the predicted class and similarity vector for each image in that folder, followed by a brief summary that specifies the number of images that were classified to each class and the average similarity vector.

### Splitting the Dataset Into Training and Test Data

When testing an image classifier, the user can determine the number of images that are used for testing and the number of images used for training. By default, 75% of the images of each class are used for the training, and the remaining 25% are used for testing. The user can change this ratio by using the "-r" option. For example, "-r0.4" allocates 40% of the images for testing, and 60% for training. The allocation of the images to training and test sets is performed in a random fashion. Users can repeat the test with several different random splits in a single command by specifying the "-n" option, followed by the requested number of splits.

*wndchrm *also allows the user to set the number of training images per class. This can be done using the "-i" option, followed by the requested number of training images per class. The remaining images are used for testing, unless the "-j" option is used in a similar fashion to set the number of test images per class. It should be noted that "-i" and "-j" options override the "-r" value. If only one of these options is specified, and "-r" is also used, the number of training or test images per class (the one that is not determined by "-i" or "-j") will be determined by the "-r" value. We suggest using a fixed number of training images per class ("-i") when generating similarity matrices.

Users can also use different feature files (generated by using *wndchrm*'s "train" command) for testing and training, so that instead of splitting a single dataset into training and test images, one dataset is used entirely for training while a second dataset is used for testing. This can be done by simply specifying two full paths to image feature files. If two files are specified, the first will be used for training and the second for testing.

### Changing the Number of Image Features

Since *wndchrm *is a multi-purpose tool designed to handle many different image datasets, it uses very many different image features. However, for a given dataset, not all image features are assumed to be equally informative, and some of these features are expected to represent noise. By default, only the 0.15 images features with the highest Fisher scores are used. Users who wish to change this setting can specify the "-f" option in the command line, followed by the requested portion of the image features to be used. Changing this value can affect the performance of the image analysis since in some datasets more image features may be informative, so that using more features can contribute to the discrimination between the classes. On the other hand, in other datasets only few of the image features provide discriminative information, and using the non-informative features can add confusion and degrade the efficacy of the analysis. Since image features are weighed by their informativeness, the effect of noisy features is expected to be lower than the effect of more informative features. However, if very many non-informative image features are used, their large number can be weighed against their low Fisher scores, leading to an undesirable degradation of the performance. Therefore, the threshold for non-informative features needs to be determined operationally for each type of data, and the 0.15 threshold is only a starting point.

### Image Tiling

In some cases it may be useful to divide large images of tissues or cells into several equal-sized tiles. For example, it has been demonstrated that when each image captures very many cells, dividing the image into tiles can in some cases provide better analysis than applying a first step of global-thresholding cell segmentation [22]. Another advantage is that using more tiles can improve the effectiveness of the Fisher scores assigned to the image features, which are expected to improve as the size of the dataset gets larger. Using *wndchrm*, this can be done by specifying the "-t" option followed by the square root of the desired number of tiles. For instance, "-t3" divides each image into 3 × 3 tiles.

If segmentation of the subjects (e.g., cell segmentation, bone segmentation, etc) is required, the user has to apply a first step of segmentation using a designated utility. The output of the utility (the segmented subjects) can be used as input for *wndchrm*, rather than the original images.

### Using Multiple Processors

As discussed earlier in the paper, response time for computing the image features can be reduced substantially by using several *wndchrm *instances running simultaneously on different processors. In order to use this feature, *wndchrm *should be started with the "-m" option. This writes the computed feature values of the images to .sig files, and checks for the existence of these files to avoid recomputing image features that have already been computed.

The purpose of the .sig files is to store the feature values of an image or image tile. I.e., *wndchrm *creates a .sig file for each tile that it processes, so that the number of .sig files of each image is equal to the number of tiles each image is divided into (one by default). If *wndchrm *finds an existing .sig file, it avoids recomputing these features and moves on to the next tile. Since the different instances of *wndchrm *communicate using the .sig files, different machines that have access to the same disk space can effectively process the same dataset simultaneously without wasting their CPU resources by processing the same tiles. To utilize this feature, the user should make sure that the number of running instances of *wndchrm *is equal to the number of available cores. For instance, a quad-core machine will be most effective if four instances of *wndchrm *run simultaneously. Since *wndchrm *does not automatically determine the number of available cores, the user should manually execute the desired number of *wndchrm *instances (usually by repeating the same command line).

In order to run *wndchrm *as a background process, the user should add an ampersand (&) at the end of the command line. This allows the user to start several instances of wndchrm using the same terminal window, and then close the window without stopping these processes.

This feature can also be highly useful in cases where data (classes or images) are being added to an existing dataset. In these cases, using the "-m" option will make wndchrm skip the images that have already been computed, so that the response time for generating the feature file that contains the new data becomes significantly shorter.

### Using the Large Feature Set

While the smaller feature set consists of 1025 features, a user can use the larger feature set of 2659 image content descriptors. The larger set of image features can be more informative, but also requires the sacrifice of more computational resources, which leads to a slower response time. The image features extracted from each transform in the smaller and larger feature sets are specified in Table 1.

In order to use the large feature set, the user can use the "-l" option in the command line. For example, the following command line will compute the large set of image features:

% wndchrm train -l/path/to/images/path/to/feature_file.fit

### Using Color Features

When color information is available, *wndchrm *can be set to use color information by using the "-c" option in the command line. If this option is specified, *wndchrm *first applies a color transform by classifying each pixel into one of 16 color classes using fuzzy logic-based modeling of the human perception of colors [23], and then assigning each pixel with an intensity value based on the relative wavelength of the classified color, normalized to [0,255] interval. I.e., pixels classified as red are assigned with 0, pixels classified as violet are assigned with 255, and pixels classified as other colors are assigned with values between 0 and 255, based on their relative wavelength. After transforming the color image into a grayscale matrix, all image features described in Table 1 are extracted from this transform.

Additionally, each pixel of the raw image is separated into its Hue, Saturation, and Value components, and the features listed in Table 1 are extracted from the hue values. The same features, except from edge features, object features, and Gabor filters are computed on the Fourier and Chebyshev transforms of the hue values. Although the hue component does not necessarily represent the actual color that the human eye perceives, it provides an objective quantification of color based on physical measurements performed by image acquisition machinery.

### Other Command-line Options

In addition to the command line options described above, the user can use the following options: -w: Use simple Weighted Nearest Neighbor instead of the WND method described by Equation 1. If this option is used, the distance of a feature vector *x *from a certain class *c *is the shortest Euclidean distance between the given sample and any training sample of that class, as defined by Equation 3

(3) $$d_{x,c}=\underset{t\in T_{c}}{\min}[\sum_{f=1}^{\left|x\right|}W_{f}{\left(x_{f}-t_{f}\right)}^{2}],$$

where *T*_*c *_is the training set of class *c*, *t *is a feature vector from *T*_*c*_, |*x*| is the length of the feature vector *x*, *x*_*f *_is the value of image feature *f *in the feature vector *x*, and *W*_*f *_is the Fisher score of feature *f*.

-d: Used for downsampling the images to N percents of their original size before computing the image features. For instance, specifying "-d50" will downsample the image by 50%. The default value for this option is 100 (no downsampling). This option can be used to accelerate the computation process of datasets that contain large images.

-q: Normally, when testing the accuracy of a certain classifier, a correct prediction is when the closest class is also the ground truth class of the tested sample. However, in some cases a user might want to consider a prediction as correct if one of the *several *closest classes is the ground truth class. For instance, if "-q5" is specified in the command line, a prediction will be considered correct if one of the closest five classes (as determined by the classifier) is the ground truth class of the given sample. The default value for this option is 1.

-N: Defines the number of classes. E.g., if a certain dataset has 100 different classes, and the option "-N25" is specified in the command line, only the first 25 classes (in an alphabetical order) will be included in the analysis, and the last 75 classes will be ignored. If this option is not specified, all classes in the dataset are used.

-v: In some cases, users might want to export or import the weights (determined using the Fisher scores) that are assigned to each feature. This can be done by using the "-v" options, followed by the requested operation and the path to a weight vector file. This gives the four following options:

-vw/path/to/weight_vector_file – exports the weights to a file.

-vr/path/to/weight_vector_file – reads the weights from a file.

-v-/path/to/weight_vector_file – compute the Fisher scores and then subtract the values in the file from the computed weights.

-v+/path/to/weight_vector_file – compute the Fisher scores and then add the values in the file to the computed weights.

-s: Eliminates the messages *wndchrm *prints to the screen and making it less verbose when it runs.

-h: Shows brief instructions and few examples for using *wndchrm*.

### Reports

An important feature of *wndchrm *is the exposition and visualization of the results in the form of reports that can be distributed and viewed using a standard web browser. This can be achieved by specifying the "-p" option and a full path to an html file. For example, a report file "report.html" is generated by using the following command:

% wndchrm test -p/path/to/feature_file/path/to/report.html

Each report includes the classification accuracy, confusion matrix, and similarity matrix of each random split into training and test data, as well as the averaged values for all splits. The number of splits can be determined by using the "-n" option. For instance, if the user specifies "-n5" in the command line, the classifier will be tested five times such that each run uses a different random split of the data into training and test images. The report also includes the average accuracy, confusion matrix and similarity matrix of the five runs. This feature can be important when the size of the test set is small, so that averaging different splits of the data can provide a more accurate measurement of the performance of the classifier. The report also lists the image features that were used for the classification along with their assigned Fisher scores, which provide information regarding the discriminative power of each feature.

In addition to similarity and confusion matrices, the report also shows the classification of each individual image. This includes the similarity values of the image to each of the classes, and its most similar training image. Specifying the most similar training image for any test image can be useful for manually checking the misclassified images and getting better intuition about how the images are being classified. In some cases it can also be used for finding flaws in the dataset.

**Figure 2:**
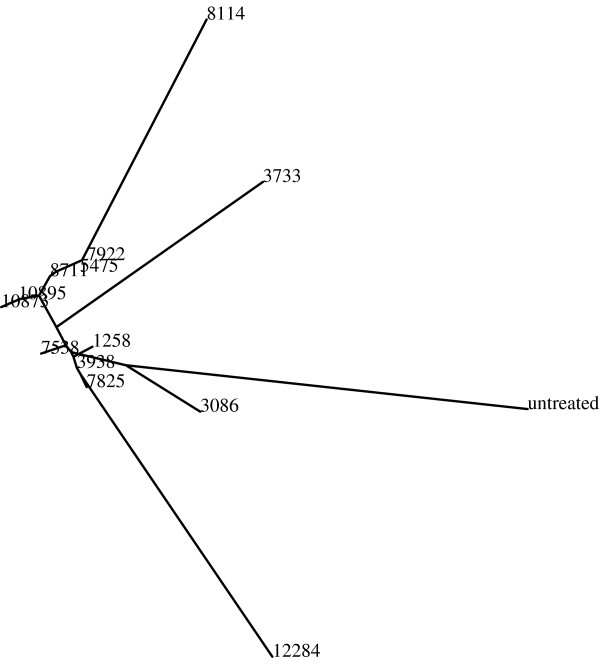
**An example phylogeny**. The phylogeny shows the visual similarities of 16 classes, where each class is a set of 25 images of Drosophila cells with single gene knockdown. The gene classes are specified by their CG numbers. As can be learned from the phylogeny, the resulting phenotypes when knocking down genes CG10895 and CG10873 (which have a substrate-kinase relationship) are very similar to each other, while the phenotypes of gene CG12284 (cell death), CG3733 (unknown function) and untreated cells are different from the other cells.

If the "-p" option is followed by a path to the root folder of phylip 3.65 or 3.67 [20], the report also features a phylogeny of the class similarities using the similarity values described earlier in the paper. The image of the phylogeny is a postscript (.ps) file, which is copied to the same folder of the report file. If *ImageMagick *is also installed on the system, a jpg image of the phylogeny will be generated and added to the html page of the report. A phylogeny can be useful, for instance, for reconstructing biological pathways by visualizing the similarities between images of different phenotypes [24]. Figure 2 shows an example of a phylogeny that visualizes phenotype similarities of Drosophila cells with single gene knockdowns.

In some cases users may want to export the values computed by *wndchrm *for further processing using external tools such as spreadsheets or statistical analysis software. This can be achieved by specifying the "+" character after the "-p" switch. If "+" is specified, *wndchrm *exports the similarity and confusion matrices in tsv (tab separated) format, which is readable by most data processing tools. The files are exported into a folder called "tsv", created in the folder of the report. If the user wishes to generate the tsv files and the phylogeny in the same command, the path to phylip should follow the "+" (e.g., -p+/path/to/phylip-3.67).

If the class names are numeric values, the report also shows the Pearson correlation between the actual and predicted values, such that a predicted value is determined by interpolating the values of the two nearest training samples that do not belong to the same class, as described by Equation 4,

(4) $$V=\left(\frac{V_{1}}{d_{1}}+\frac{V_{2}}{d_{2}}\right)/\left(\frac{1}{d_{1}}+\frac{1}{d_{2}}\right)$$

where *V *is the resulting predicted value, and *d*_1_, *d*_2 _are the distances from the nearest two samples *V*_1 _and *V*_2_, such that *V*_1 _and *V*_2 _belong to two different classes. The distances *d*_1 _and *d*_2 _are computed by Equation 1.

## Using the Source Code

All features described in the previous section can be integrated into existing or newly developed software products. A detailed example of how the function calls are used can be found in the file "wndchrm.cpp", which implements the command line user interface.

The basic data structure of the code is the class "TrainingSet", which is implemented in the file "TrainingSet.cpp". This class can be used to compute image features of a given dataset, test a dataset, generate reports, and more. Generally, computing image features for a given dataset of images can be implemented by first creating an instance of the class "TrainingSet", calling the method "LoadFromDir" to compute the image features of a dataset, and then saving to a file as described by the following code:

#include "TrainingSet.h"

   TrainingSet *ts;

   ts = new TrainingSet();

   ts->LoadFromDir();

   ts->SaveToFile();

A dataset can be tested by first loading a file of computed image features, splitting it into training and test sets, normalizing the values, computing Fisher scores using the training samples, and then testing the classification accuracy using the samples allocated for the test set. This sequence of operations is summarized by the following code:

TrainingSet *ts,*train,*test;

ts = new TrainingSet();

ts->ReadFromFile();

ts->split(train, test)

train->normalize();

train->SetFisherScores();

train->Test(test);

It is important that the "normalize()" method is called *before *the Fisher scores are computed (using "SetFisherScores"). The reason is that the different image features have different ranges of values. In order to use the differences between the values of each feature as described in Equation 1, the values should be normalized such that all image features have the same range, which is set to [0,1]. The normalization of a certain feature *f *of a specific image *i *is performed using Equation 5

(5) $${\hat{f}}_{i}=[\max(m|m\in T_{f})-\min(n|n\in T_{f})]\cdot (f_{i}-\min(n|n\in T_{f})),$$

where ${\hat{f}}_{i}$ is the normalized value of the feature *f *of image *i*, *f*_*i *_is the original feature value, and *T*_*f *_is the set of all values of the feature *f*, computed from all images in the dataset.

In some cases, users might want to add to the *wndchrm *utility more image features that are necessary for their specific needs. This should be done by modifying the file "signatures.cpp", which is the implementation of the class "signatures". The class has two higher-level methods for computing image features: "ComputeGroups" for computing the larger set of image features, and "compute" for computing the smaller set. Functions that compute new image features can be called from inside these methods, and the computed values can be added simply by using the "Add" method.

## Results and Discussion

**Figure 3:**
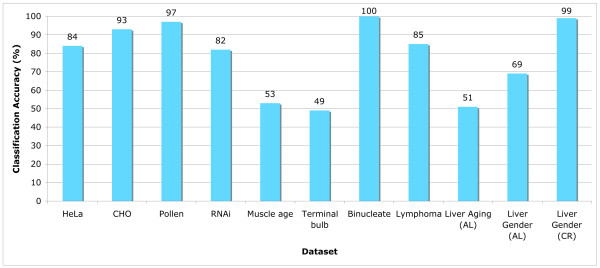
**Classification accuracy using iicbu-2008**. As the graph shows, some of the image datasets were classified with very high accuracy, such as Pollen, Binucleate, and Liver age (gender). Other datasets such as HeLa, Lymphoma and RNAi were classified in accuracy of 80–85%, and the datasets Muscle Age, Terminal Bulb and Liver Aging (age) provided classification accuracy of around 50%.

The efficacy of the proposed image analysis utility was tested using iicbu-2008 benchmark suite of biological image datasets [25], which includes biological images of different subjects such as organelles, cells, tissues, and full organisms using different magnifications and different types of microscopy. The number of classes, number of images, microscopy, image formats, and image sizes are specified in Table 2. This benchmark suite represents a broad range of real-life biological imaging problems. The performance of *wndchrm *on each of the datasets is described by Figure 3. Comparing some of these performance figures to the reported performance of application-specific image classifiers shows that *wndchrm *is favorably comparable, as can be learned from Table 3. The informativeness of the different image features and their importance for each of these datasets is described in [5]. The benchmark suite of iicbu-2008, as well as sample images of each dataset, are available for free download at [26].

**Table 2:** Test Datasets

Test Datasets				
Dataset	# of classes	# of images	# Image format	Microscopy

Pollen	7	630	25 × 25 8 bit TIFF	Phase contrast
RNAi	10	200	1024 × 1024	16 bit TIFF Fluorescence
C. elegans muscle age	4	252	1600 × 1200 16 bit TIFF	Fluorescence
Terminal bulb aging	7	970	300 × 300 16 bit TIFF	DIC
Binucleate	2	40	1280 × 1024 16 bit TIFF	Fluorescence
Lymphoma	3	375	1388 × 1040 32 bit TIFF (color)	Brightfield
Liver age	18	1500	1388 × 1040 32 bit TIFF (color)	Brightfield
2D HeLa	10	860	382 × 382 16 bit TIFF	Fluorescence
CHO	5	340	512 × 382 16 bit TIFF	Fluorescence

Number of classes, number of images, format, size and microscopy of the datasets.

**Table 3:** Comparison of wndchrm accuracy to application-specific classifiers

Classification Accuracy			
Dataset	Benchmark algorithm	Benchmark accuracy (%)	Accuracy of wndchrm (%)

Hela	[29]	83	86
Pollen	[30]	79	96
CHO	[31]	87	95

HeLa dataset [32], consists of fluorescence microscopy images of HeLa cells using 10 different labels, Pollen dataset [30], shows geometric features of pollen grains, and the CHO dataset [31], features fluorescence microscopy images of different sub-cellular compartments.

While *wndchrm *demonstrated convincing performance on these benchmark datasets, it is important to note that since there is no "typical" biological experiment, there is also no defined scale for the expected accuracy of the classifier. The classification accuracy is influenced by very many factors. These include the number of classes and the number of images per class, but also parameters that are more difficult to quantify such as the quality of the images, the consistency of the images within each class and the differences between the classes. To improve the performance of a classifier, one can add more images to the dataset and increase the size of the training set. Another way to improve the classification accuracy is to manually curate low quality images or images that are inconsistent with the other images in the class. Finally, highly similar classes can be merged into one class, and then another classifier can be built to separate the images classified into the merged class. This technique can improve the classification accuracy because the second classifier assigns higher Fisher scores to the features that classify between these specific classes.

**Figure 4:**
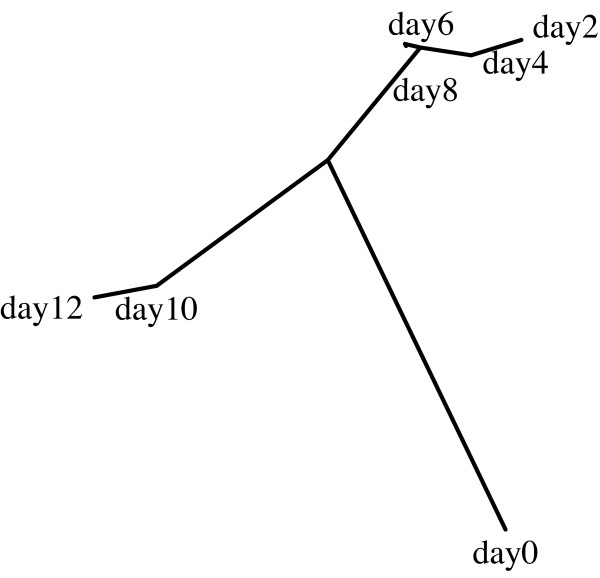
**Phylogeny of the worm terminal bulb aging**. The phylogeny that was automatically generated by wndchrm shows a class order that is in agreement with the chronological ages.

Many biological problems focus not only on the classification of different sets of images, but also on assessing the similarities between the different classes. The similarities between the classes are reflected by the similarity table in the report of the "test" command, and can be visualized using a phylogeny generated by the phylip package. For example, Table 4 shows the similarity values between different classes of C. elegans terminal bulb images, such that each class of images was taken at a different age of 0, 2, 4, 6, 8, 10, or 12 days. As the table shows, the similarities between the different classes correspond to the age differences. Visualizing these data using a phylogeny provides an ordered list of the different ages, shown by Figure 4. This order, inferred automatically by *wndchrm*, is in agreement with the chronological ages of the worms. The only exception is day 0, in which the worms still grow, and therefore expected to be significantly different from adult worms. An interesting observation is the large difference between day 8 and day 10. This experiment demonstrates that *wndchrm *can automatically deduce the continuos nature of aging by measuring the similarities between images taken at different ages. Example terminal bulb images as well as a downloadable archive of the entire set can be found at [26].

**Table 4:** Similarity matrix of the terminal bulb worm aging

Similarity Matrix							
	day 0	day 2	day 4	day 6	day 8	day 10	day 12

day 0	1.00	0.66	0.63	0.59	0.63	0.55	0.58
day 2	0.68	1.00	1.00	0.99	0.95	0.76	0.65
day 4	0.64	0.93	1.00	1.00	0.97	0.82	0.71
day 6	0.55	0.84	0.96	1.00	1.00	0.89	0.75
day 8	0.55	0.77	0.89	0.93	1.00	0.84	0.71
day 10	0.53	0.52	0.70	0.63	0.77	1.00	0.98
day 12	0.49	0.48	0.65	0.58	0.68	0.94	1.00

Each class consists of microscopy images of the terminal bulb of C. elegans nematodes taken at ages 0, 2, 4, 6, 8, 10, 12 days. As the table shows, the similarity values are higher for neighboring age groups. The dataset can be downloaded at .

Another example uses automatically acquired microscopy images of drosophila cells such that each class contains images of the resulting phenotype of a single gene knockdown. Table 5 shows the genes and the similarity values that reflect the visual similarities between the different phenotypes. This experiment uses *wndchrm *in order to measure and quantify phenotype similarities for the purpose of reconstructing biological pathways and finding genes that are part of the same cellular mechanisms. This type of analysis can be used for finding similarities between genes based on the phenotypes that they produce, in contrast to finding similarities between genes by analyzing their sequences using methods such as BLAST. A similar analysis was used to produce the phylogeny of Figure 2. This dataset is publicly available at [26].

**Table 5:** Similarity matrix of 10 different phenotypes of drosophila cells

Similarity Matrix										
	CG10873	CG12284	CG1258	CG17161	CG3733	CG3938	CG7922	CG8114	CG8222	CG9484

CG10873	1.00	0.74	0.85	0.84	0.96	0.96	0.83	0.89	0.92	0.90
CG12284	0.61	1.00	0.75	0.67	0.68	0.69	0.68	0.79	0.77	0.68
CG1258	0.82	0.82	1.00	0.94	0.87	0.83	0.73	0.92	0.92	0.90
CG17161	0.78	0.75	0.85	1.00	0.84	0.81	0.72	0.78	0.84	0.86
CG3733	0.97	0.72	0.91	0.90	1.00	0.95	0.81	0.90	0.94	0.88
CG3938	0.99	0.82	0.88	0.86	0.95	1.00	0.93	0.89	0.98	0.94
CG7922	0.93	0.80	0.80	0.80	0.89	0.96	1.00	0.78	0.92	0.92
CG8114	0.82	0.86	0.93	0.83	0.80	0.80	0.69	1.00	0.89	0.82
CG8222	0.92	0.88	0.96	0.90	0.94	0.93	0.86	0.95	1.00	0.91
CG9484	0.72	0.74	0.87	0.81	0.78	0.81	0.79	0.84	0.83	1.00

Each class is a set of light microscopy images (60×) of drosophila cells with single gene knockdowns. The genes are the class names. The dataset is available at .

Since *wndchrm *has been found useful for a relatively wide range of biological imaging problems, we believe that this utility can be useful for researchers who wish to apply biological image analysis to high content screening or other biological experiments that involve high volumes of image data. The source code can be integrated into other software products using the source files or the "libimfit" library, but is also wrapped as a command line utility that can be easily used by researchers who have basic computer skills and no previous knowledge in programming. We therefore advise investigators to first use *wndchrm *before taking the costly challenge of developing application-specific image classifiers.

Future plans include the addition of three-dimensional image features, and also analysis of the images for finding the more informative areas and differentiating them from other areas that produce a weaker signal.

Additionally, *wndchrm *will be able to read images directly from OME image server, so that all image formats supported by OME will be also supported by *wndchrm*.

Full source code is available for free download as part of OME [1,2] software suite at [27], or as a "tarball" at [28].

## Conclusion

The availability of biological image acquisition systems and storage devices allows research that is based on high content screening of vast pipelines of biological images. However, while image analysis has been becoming increasingly important in biological experiments, machine vision algorithms are usually developed by pattern recognition and signal processing experts, and biologists often do not have the knowledge and resources to develop these algorithms and software tools.

Here we present a utility that can be used by experimental biologists, and has been shown to be effective for various actual biological experiments using real-life biological data. While advanced users can embed the code and libraries into their own software tools, researchers who have basic computer skills can easily use the application as a command line utility. This can assist experimental biologists in obtaining effective image analysis capabilities, yet without the sacrifice involved in designing, developing, and testing new software tools.

## Authors' contributions

LS developed the described software and prepared the manuscript. NO, TM, JJ, LS and IG developed an earlier MATLAB implementation of the smaller feature set and the WND feature classification algorithm. DME provided data, performed software testing, edited the manuscript, and advised on usability issues.
